# Review of the bamboo-feeding leafhopper genus *Neomohunia*, with descriptions of two new species from China (Hemiptera, Cicadellidae, Deltocephalinae, Mukariini)

**DOI:** 10.3897/zookeys.790.26130

**Published:** 2018-10-15

**Authors:** Qiang Luo, Lin Yang, Xiang-Sheng Chen

**Affiliations:** 1 Institute of Entomology, Guizhou University, Guiyang, Guizhou, 550025, PR China Guizhou University Guiyang China; 2 The Provincial Special Key Laboratory for Development and Utilization of Insect Resources of Guizhou, Guizhou University, Guiyang, Guizhou, 550025, PR China Guizhou University Guiyang China; 3 Guizhou Key Laboratory for Plant Pest Management of Mountainous Region, Guizhou University, Guiyang, Guizhou, 550025, PR China Guizhou University Guiyang China

**Keywords:** Homoptera, morphology, Oriental region, taxonomy

## Abstract

The bamboo-feeding leafhopper genus *Neomohunia* Chen & Li, 2007, is reviewed to include three species: *N.longispina***sp. n.**, *N.pyramida* (Li & Chen, 1999), and *N.sinuatipenis***sp. n.** The generic characteristics are redefined and the new species are described and illustrated. A key to species based on male genitalia is also provided.

## Introduction

Chen and Li (2007) established the Chinese bamboo-feeding leafhopper genus *Neomohunia* (Cicadellidae: Deltocephalinae: Mukariini) for *Mohuniapyramida* Li & Chen, 1999 (type species). The genus belongs to the tribe Mukariini based on body medium sized, with orange, brown and reddish orange markings dorsally; head moderately produced; ocelli distant from eyes; frontoclypeus strongly convex basally, depressed apico-medially, without median carina. Forewing venation obscure except near apex, with four apical cells and appendix well developed.

In this paper, two new species: *N.longispina* sp. n. and *N.sinuatipenis* sp. n., from China are described and illustrated. A key based on male genitalia to distinguish males of all three included species is given.

## Materials and methods

Terminology used for morphological and genital characters follow Li et al. (2011) and Zahniser and Dietrich (2013). Leg chaetotaxy follows Dietrich (2005). All specimens were collected by sweep net, dry male specimens were used for the description and illustration. External morphology was observed under a stereoscopic microscope and characters were measured with an ocular micrometer. Measurements are given in millimeters; body length is measured from the apex of the head to the apex of the forewing in repose. Habitus photographs were taken using a KEYENCE VHX-1000 system. The genital segments of the specimens examined were macerated in 10% NaOH and drawn from preparations in glycerin jelly using a Leica MZ 12.5 stereomicroscope. The photographs and the illustrations were scanned with Canon CanoScan LiDE 100 and imported into Adobe Photoshop CS5 for plate composition and labeling.

The type specimens examined are deposited in the Institute of Entomology, Guizhou University, Guiyang, Guizhou Province, China (**IEGU**) and the Natural History Museum, UK (**NHMUK**).

## Taxonomy

### Genus Neomohunia

Taxon classificationAnimaliaHemipteraCicadellidae

Chen & Li, 2007

#### Type species.

*Mohuniapyramida* Li & Chen, 1999, by original designation.

#### Diagnosis.

The genus is separated from other similar genera of Mukariini by crown rounded to face, without apical transverse marginal carina; frontoclypeus strongly convex dorsally, depressed ventro-medially; male pygofer with one or two processes at caudal apex; subgenital plate with numerous macrosetae laterally; aedeagus with pair of spinous processes arising from base, with or without a single ventral basal medial process.

#### Description.

Medium-sized, delicate leafhoppers; with orange, brown and reddish orange markings dorsally including reddish medial longitudinal stripe on head and pronotum.

**Figures 1–13.**
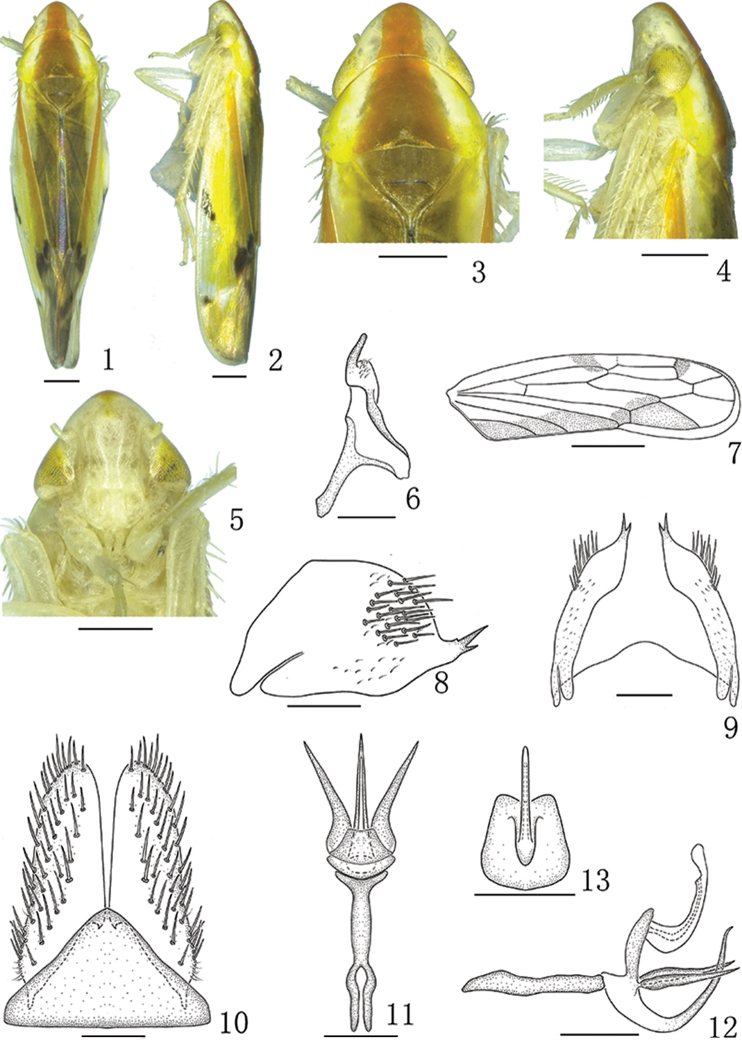
*Neomohunialongispina* sp. n., male **1** Male habitus, dorsal view **2** Male habitus, lateral view **3** Head and thorax, dorsal view **4** Head and thorax, lateral view **5** Face **6** Style, dorsal view **7** Forewing **8** Male pygofer, lateral view **9** Male pygofer, ventral view **10** Valve and subgenital plate, ventral view **11** Connective and aedeagus, dorsal view **12** Connective and aedeagus, lateral view **13** Shaft and preatrium, caudal view. Scale bars: 0.5 mm (**1–5, 7**); 0.2 mm (**6, 8–13**).

**Figures 14–25.**
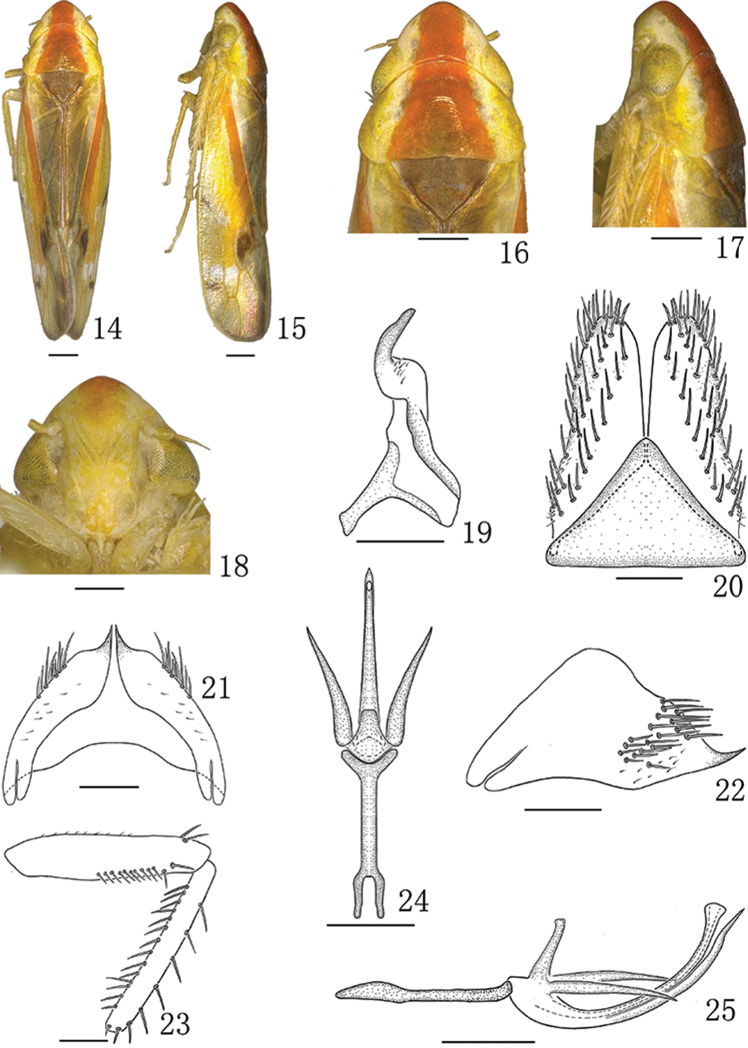
*Neomohuniapyramida* (Li & Chen, 1999), male **14** Male habitus, dorsal view **15** Male habitus, lateral view **16** Head and thorax, dorsal view **17** Head and thorax, lateral view **18** Face **19** Style, dorsal view **20** Valve and subgenital plate, ventral view **21** Male pygofer, ventral view **22** Male pygofer, lateral view **23** Fore femur and tibia, anterior surface **24** Connective and aedeagus, dorsal view **25** Connective and aedeagus, lateral view. Scale bars: 0.5 mm (**14–18**); 0.2 mm (**19–25**).

**Figures 26–36.**
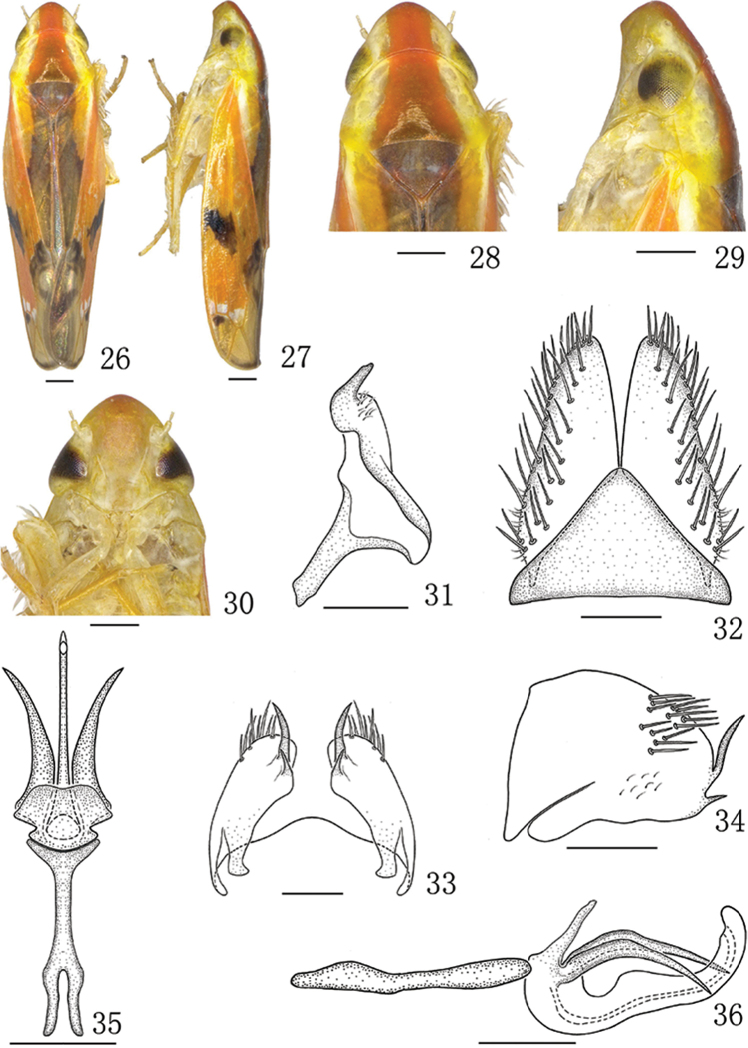
*Neomohuniasinuatipenis* sp. n., male **26** Male habitus, dorsal view **27** Male habitus, lateral view **28** Head and thorax, dorsal view **29** Head and thorax, lateral view **30** Face **31** Style, dorsal view **32** Valve and subgenital plate, ventral view **33** Male pygofer, ventral view **34** Male pygofer, lateral view **35** Connective and aedeagus, dorsal view **36** Connective and aedeagus, lateral view. Scale bars: 0.5 mm (**26–30**); 0.2 mm (**31–36**).

*Head and thorax.* Head moderately produced, apex in profile truncate (Figs 4, 17, 29). Crown slightly convex and rounded to face, without anterior marginal carina, median length subequal to width between eyes (Figs 3–4, 16–17, 28–29); coronal suture short; ocelli near crown margin, equidistant from eyes to crown apex (Figs 4, 17, 29). Face with frontoclypeus strongly convex basally, depressed apico-medially, without median carina; clypellus with lateral margins parallel; lorum broad (Figs 5, 18, 30). Pronotum broad, wider than head including eyes, with lateral margins divergent posteriorly, anterior margin strongly convex between eyes, posterior margin weakly concave (Figs 1, 3, 14, 16, 26, 28). Mesoscutum and scutellum together wider than long, transverse suture slightly curved and depressed, not reaching lateral margin (Figs 3, 16, 28). Forewing elongate, considerably longer than abdomen, with four apical cells, venation obscure except near apex, vein M_3+4_ originating from inner anteapical cell, converging toward middle of appendix; appendix well developed (Figs 1–2, 14–15, 26–27). Hind wing with four closed apical cells. Profemur with AM1 and AV1 present, intercalary row with 10 or more slender setae. Protibia with macrosetal formula 7+1 and approximately 14 macrosetae of equal length in row AV (Figure 23). Hind femur macrosetal formula 2+2+1.

*Male genitalia.* Male pygofer broad at base in lateral aspect, tapering caudally with one or two processes at caudal apex; with macrosetae ventrocaudally (Figs 8–9, 21–22, 33–34). Valve triangular (Figs 10, 20, 32). Subgenital plate nearly triangular, with numerous macrosetae laterally (Figs 10, 20, 32). Connective Y-shaped, with stem longer than arms, apex broad (Figs 11, 24, 35). Aedeagus with pair of spinous processes arising either dorsobasally on shaft or from preatrium, with or without a single ventral basal medial process; gonopore apical or subapical on dorsal surface (Figs 11–13, 24–25, 35–36). Style with articulating arm moderately long and robust, apophysis digitate, slightly laterally curved (Figs 6, 19, 31).

**Figures 37–45.**
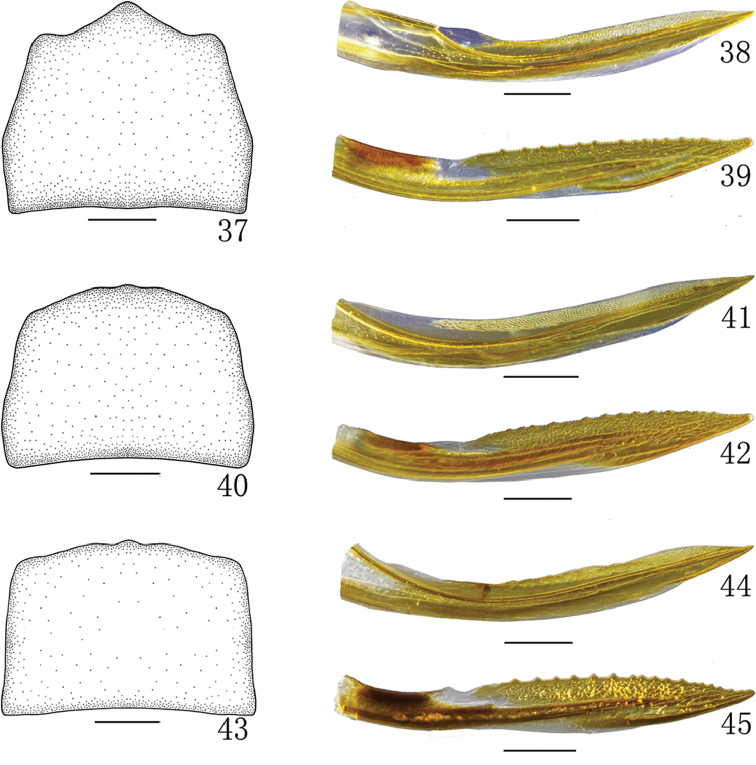
**37–39***Neomohunialongispina* sp. n., female **37** Female sternite VII, ventral view **38** First valvula, lateral view **39** Second valvula, lateral view **40–42***Neomohuniapyramida* (Li & Chen, 1999), female **40** Female sternite VII, ventral view **41** First valvula, lateral view **42** Second valvula, lateral view **43–45***Neomohuniasinuatipenis* sp. n., female **43** Female sternite VII, ventral view **44** First valvula, lateral view **45** Second valvula, lateral view. Scale bars: 0.2 mm (**37–45**).

*Female genitalia.* Sternite VII (Figs 37, 40, 43) with posterior margin strongly or slightly convex, with or without acute median tooth. First valvula (Figs 38, 41, 44) weakly curved, tapering apically with strigate sculpture extended to dorsal margin. Second valvula (Figs 39, 42, 45) broad, widest at distal two thirds, thereafter gradually tapered to acute apex; dorsal margin with numerous triangular, distinct and regular teeth; with dorsal sclerotized and hyaline region and dorsal prominence (in *N.sinuatipenis*).

#### Host plants.

Bamboo.

#### Distribution.

China (Guizhou).

### Key to species of the genus *Neomohunia* (males)

**Table:** 

1	Aedeagal shaft with a ventral medial process arising from basal one-third of shaft (Figs 24–25)	***N.pyramida* (Li & Chen)**
–	Aedeagal shaft without a medial ventral process	**2**
2	Aedeagal shaft sinuate in lateral view, with two dorsal processes arising from base (Figs 35–36)	***N.sinuatipenis* sp. n.**
–	Aedeagal shaft evenly curved in lateral view; three spinous processes arising from base of preatrium of aedeagus (Figs 11–13)	***N.longispina* sp. n.**

### Neomohunia longispina sp. n.

Taxon classificationAnimaliaHemipteraCicadellidae

http://zoobank.org/3E6AADA6-89B0-4A03-B305-6417F4F36832

Figs 1–13 , 37–39 , 48

#### Diagnosis.

The salient characteristics of the new species include the pygofer in profile with pair of small unequal spines arising directly from posteroventral margin (Figs 8–9), and the aedeagus with its shaft slightly laterally compressed, with three spinous processes arising from base of long preatrium (Figs 11–13).

#### Description.

*Measurements.* Body length (including forewing): male 5.11–5.67 mm (7 specimens); female 5.92–5.98 mm (4 specimens).

*Coloration*. Crown and pronotum pale yellow to white, with a longitudinal medial bright red band widening from apex of head to base of pronotum (Figs 1, 3). Eyes yellowish brown to brown (Figs 2, 4). Face yellow, anteclypeus, and lorum yellowish white (Figure 5). Mesoscutum and scutellum brown (Figs 1, 3). Forewing orange red to red, clavus with a longitudinal broad brown stripe along lateral margin (Figs 1, 2, 7); brachial cell with a bright red stripe along inner margin (Figs 1, 2, 7); brachial cell at apex, inner anteapical cells near m-cu3, costal margin near middle, outer apical cell near R_2+3_ with dark brown markings (Figs 1, 2, 7); outer and central anteapical cells with a pellucid spot at apex, inner apical cell slightly brown (Figs 1, 2).

*Head and thorax*. External features as in generic description with following proportions. Crown slightly shorter medially than width between eyes (0.68:1) (Figs 1, 3); coronal suture shorter than half of length of crown in median line (0.35:1) (Figs 1, 3). Pronotum slightly wider than head including eyes (1.19:1) and about 2 times longer than head (1.85: 1) (Figs 1, 3). Mesoscutum and scutellum together distinctly shorter than pronotum (0.70:1) (Figure 3). Forewing about 3 times longer than widest part (2.75: 1) (Figs 1, 2).

*Male genitalia*. Pygofer in profile with pair of small unequal spines arising directly from posteroventral margin (Figs 8–9). Aedeagus with shaft slightly laterally compressed, gonopore subapical on dorsal surface; with three spinous processes arising from base of long preatrium, two shorter lateral processes directed obliquely outwards and a longer medial process curving ventrally from base at its articulation with connective then curved dorsally and tapered to acute apex (Figs 11–13).

*Female genitalia.* Sternite VII (Figure 37) with anterior margin slightly concave; lateral margin slightly expanded at basal 1/3; posterior margin strongly convex and with acute median tooth. Ovipositor as in generic description.

#### Material examined.

Holotype: ♂, **China**: Guizhou Province, Xishui County, Sanba Nature Reserve (28°20'N, 106°12'E), 27 September 2017, Bin Yan and Nian Gong (IEGU); paratypes: 4♂♂3♀♀, same data as holotype (IEGU); 2♂♂1♀, same data as holotype (NHMUK).

#### Host plant.

Bamboo (Figure 48).

#### Distribution.

China (Guizhou Province).

#### Remarks.

This species can be distinguished from other species mainly by the unusual position of the aedeagal processes at the base of the preatrium (Figs 11–13).

#### Etymology.

The name is derived from prefix *longi* and the Latin word *spina*, which refers to the long medial process of the aedeagus.

### Neomohunia pyramida

Taxon classificationAnimaliaHemipteraCicadellidae

(Li & Chen, 1999)

Figs 14–25 , 40–42 , 46–47

Mohunia pyramida Li & Chen, 1999: 123, figs 1–10.
Neomohunia pyramida : Chen, Li and Yang 2007: 373, figs 36–46.

#### Diagnosis.

This species has a pygofer which, in profile, is more triangular with its apex tapering into a single stout spinous process, the dorsal margin sinuate (Figs 21–22), the aedeagal shaft (Figs 24–25) curved dorsally, tubular with apex blunt, and with a ventral medial process arising from basal one-third and two processes arising dorsobasally (Figs 24–25).

#### Description.

*Measurement.* Body length (including forewing): male 5.12–5.46 mm (37 specimens), female 5.81–6.07 mm (48 specimens).

External features as in *N.longispina*.

*Male genitalia.* Male genitalia as in previous species but pygofer in profile more triangular with apex tapering into a single stout spinous process, dorsal margin sinuate (Figs 21, 22). Aedeagal shaft (Figs 24, 25) curved dorsally, tubular with apex blunt, one spinous process arising ventrally from basal one-third thereafter closely appressed to shaft and then diverging at distal one-third; another processes arising on each side dorsobasally, directed caudally; gonopore apical.

*Female genitalia.* Sternite VII (Figure 40) with both anterior margin and posterior margin slightly concave. Ovipositor as in generic description.

#### Material examined.

1♂ (holotype), **China**: Guizhou Province, Suiyang County, Kuankuoshui Nature Reserve (27°58'N, 107°11'E), 28 July 1984, Zi-Zhong Li (IEGU); 5♂♂l1♀♀ (paratypes), same data as holotype (IEGU); 1♂1♀ (paratypes), same data as holotype (NHMUK). 1♂4♀♀ (paratypes), Guizhou Province, Jiangkou County, Fanjingshan National Nature Reserve (27°55'N, 108°41'E), 11 August 1984, Xiang-Sheng Chen and Mao-Fa Yang (IEGU); 2♂♂3♀♀, Guizhou Province, Suiyang County, Kuankuoshui Nature Reserve, 1 August 1984, Zi-Zhong Li (IEGU); 1♀, Guizhou Province, Guiyang City, Forest Park (26°35'N, 106°42'E), 12 June 2002, De-Yan Ge (IEGU); 1♂, Guizhou Province, Jiangkou County, Fanjingshan National Nature Reserve, 28 July 2002, Zi-Zhong Li (IEGU); 6♂♂9♀♀, Guizhou Province, Daozhen County, Dashahe Nature Reserve (28°53'N, 107°36'E), 22–23 May 2004, Xiang-Sheng Chen (IEGU); 15♂♂15♀♀, same locality, 17–24 August 2004, Xiang-Sheng Chen, Bin Zhang and Mao-Fa Yang (IEGU); 5♂♂ 4♀♀, Guizhou Province, Fanjingshan National Nature Reserve, 31 June 2004, Xiang-Sheng Chen (IEGU); 9♀♀, Guizhou Province, Suiyang County, Kuankuoshui Nature Reserve, 28 July 2014, Yan-Li Zheng (IEGU); 3♂♂2♀♀, Guizhou Province, Suiyang County, Kuankuoshui Nature Reserve, 12 July 2017, Nian Gong (IEGU).

#### Host plant.

**Figures 46–48.**
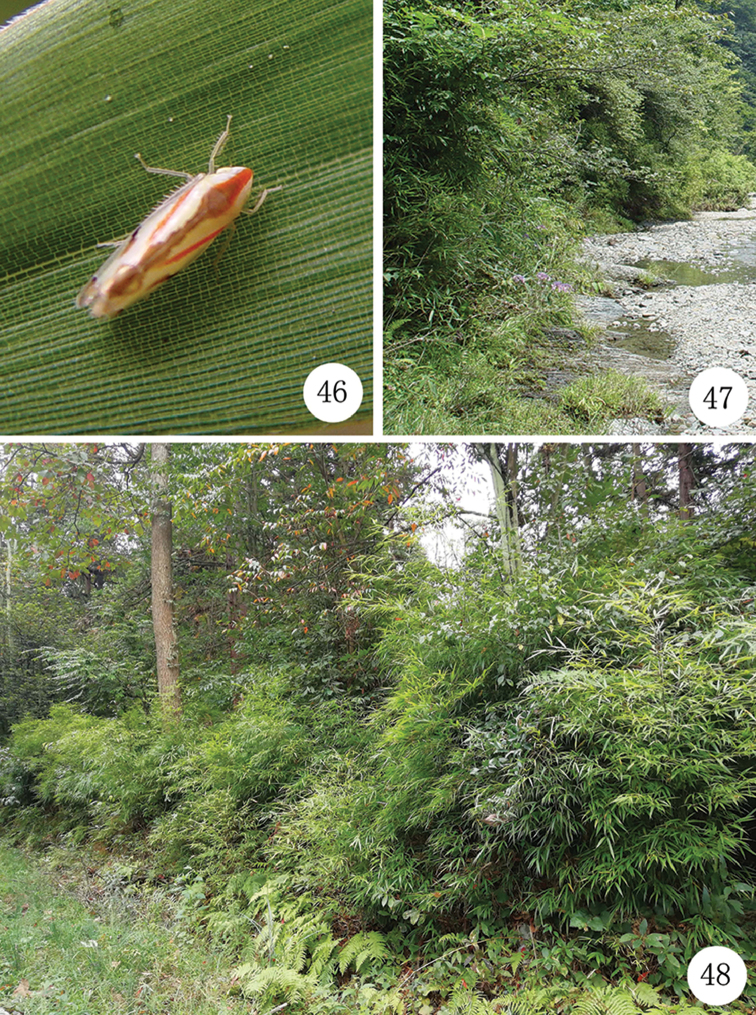
**46***Neomohuniapyramida* (Li & Chen, 1999) resting on a leaf of host plant (bamboo) **47** The habitat photo of *N.pyramida* (Li & Chen, 1999) (Guizhou Province, Daozhen County, Dashahe, 19 August 2004, photography by Xiang-Sheng Chen) **48** The habitat photo of *Neomohunialongispina* sp. n. (Guizhou Province, Xishui County, Sanba Nature Reserve, 27 September 2017, photography by Nian Gong).

Bamboo (*Qiongzhueacommunis* and *Fargesiaspathacea*) (Figs 46, 47).

#### Distribution.

China (Guizhou Province).

#### Remarks.

We re-examined the type specimens of this species and found that there were some inaccuracies in original figures in Chen et al. (2007), e.g., the style was damaged. Hence, we have redrawn the species and provide digital images of the male adult. The species resembles *N.sinuatipenis* sp. n. but differs from the later by the aedeagal shaft being tubular with a blunt apex and with a ventral medial process arising from the basal one-third of shaft. Additionally, the gonopore is apical (Figs 24, 25).

### Neomohunia sinuatipenis sp. n.

Taxon classificationAnimaliaHemipteraCicadellidae

http://zoobank.org/709ECD56-9397-4B5F-80B7-A5915E1CDFF6

Figs 26–36 , 43–45 , 49–51

#### Diagnosis.

The characteristics of the new species include the following: pygofer with ventro-posterior angle produced into one short and a long process arising directly from posteroventral margin (Figs 33, 34); aedeagus with shaft laterally compressed, sinuate in lateral view, with a pair of long spinous processes arising dorsobasally, curved caudoventrally (Figs 35, 36).

#### Description.

*Measurements*. Body length (including forewing): male 4.87–5.30 mm (10 specimens); female 5.51–5.84 mm (8 specimens).

External features as in *N.longispina* but body slightly smaller.

*Male genitalia*. Male pygofer as in *N.longispina* but pygofer with ventroposterior angle produced into one short and a long process arising directly from posteroventral margin, the shorter process directed posteriorly and the longer one directed dorsally (Figs 33–34). Aedeagus simple, with shaft laterally compressed, sinuate in lateral view, with a pair of long spinous processes arising dorsobasally, curved caudoventrally; gonopore subapical on dorsal surface (Figs 35–36).

*Female genitalia.* Sternite VII (Figure 43) as in *N.pyramida*. Ovipositor as in generic description.

#### Material examined.

Holotype: ♂, **China**: Guizhou Province, Duyun City, Doupengshan (26°22'N, 107°23'E), 18 August 2016, Jian-Kun Long (IEGU); paratypes: 1♂4♀♀, same locality, 24 September 2016, Qiang Luo and Ya-Lin Yao (IEGU); 1♂, Guizhou Province, Leishan County, Leigong Mountain (26°22'N, 108°10'E), 7 September 2014, Xiang-Sheng Chen (NHMUK); 7♂♂4♀♀, Guizhou Province, Anlong County, Xianheping (24°59'N, 105°37'E), 28 August 2012, Jian-Kun Long (IEGU).

#### Host plant.

**Figures 49–51.**
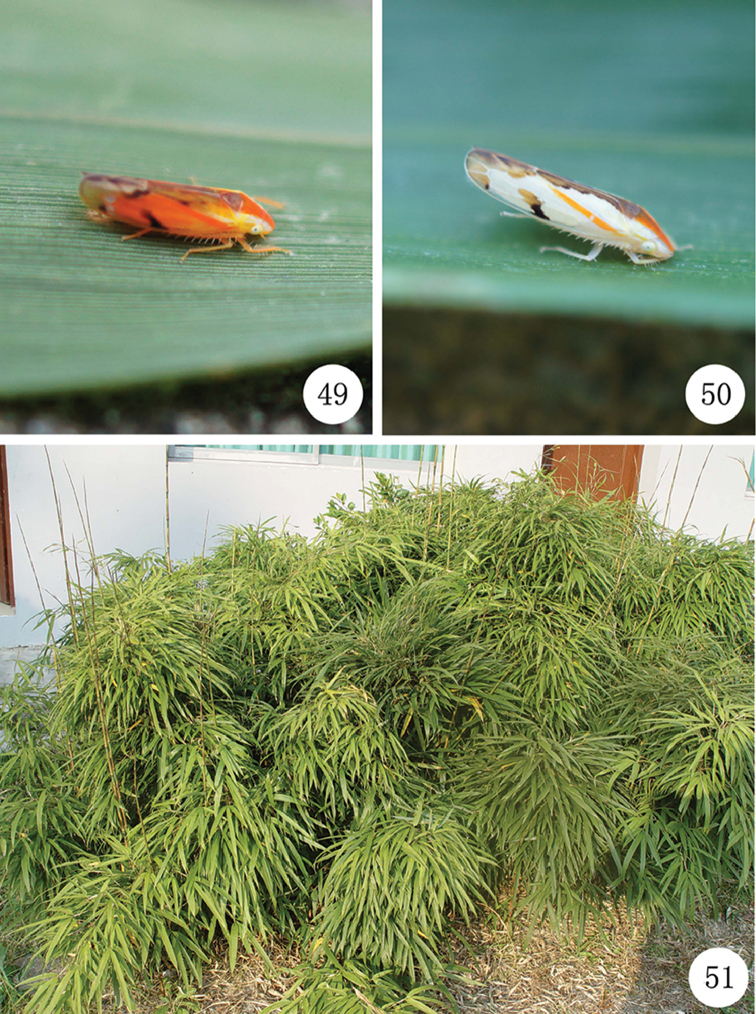
*Neomohuniasinuatipenis* sp. n. **49***N.sinuatipenis* sp. n. resting on a leaf of host plant (bamboo)(male) **50***N.sinuatipenis* sp. n. resting on a leaf of host plant (bamboo)(female) **51** The habitat photo of *N.sinuatipenis* sp. n. (Guizhou Province, Leishan County, Leigong Mountain, 7 September 2014, photography by Xiang-Sheng Chen).

Bamboo (Figs 49–51).

#### Distribution.

China (Guizhou Province).

#### Remarks.

The new species is similar to *N.pyramida* (Li & Chen, 1999), but differs in the aedeagal shaft being sinuate in lateral view, with two dorsal processes arising from base; the gonopore is subapical (Figs 35, 36).

#### Etymology.

The name is derived from the Latin words *sinuosus* and *penis*, which refers to the sinuate aedeagal shaft in lateral view (Figure 36).

## Discussion

Species of *Neomohunia* are distinctly marked leafhoppers, mainly with orange, brown and reddish orange markings dorsally including a reddish medial longitudinal stripe on the head and pronotum. In the male genitalia they can be distinguished by the aedeagus with pair of spinous processes arising from base. All are very similar in coloration and difficult to distinguish externally, but can be easily separated from other species by the structure of male genitalia: (1) aedeagal shaft evenly curved in lateral view; three spinous processes arising from base of preatrium of aedeagus in *N.longispina* sp. n.; (2) aedeagal shaft with a ventral medial process arising from basal one-third of shaft in *N.pyramida*; (3) aedeagal shaft sinuate in lateral view in *N.sinuatipenis* sp. n.

As a result of our investigation in the field, members of *Neomohunia* were found feeding exclusively on some native bamboos, with many specimens collected from the beginning of May to the end of September in Guizhou province. So far, there are no collection records in other zoogeographic regions or on other plants in China, which may suggest that the distribution and host of *Neomohunia* species are very limited. More precise ecological records are needed.

## Supplementary Material

XML Treatment for Neomohunia

XML Treatment for Neomohunia longispina

XML Treatment for Neomohunia pyramida

XML Treatment for Neomohunia sinuatipenis
